# Zebrafish as a model for human epithelial pathology

**DOI:** 10.1186/s42826-025-00238-6

**Published:** 2025-02-03

**Authors:** Ahmed Abu-Siniyeh, Moayad Khataibeh, Walid Al-Zyoud, Majed Al Holi

**Affiliations:** 1 Department of Medical Laboratory Sciences, School of Science, The University of Jordan, Amman, Jordan; 2 Department of Medical Laboratory Sciences, Faculty of Science, Al-Balqa Applied University, As Salt, Jordan; 3 Department of Biomedical Engineering, School of Applied Medical Sciences, German Jordanian University, Amman, 11180 Jordan; 4 Cell Therapy Center, The University of Jordan, Amman, Jordan

**Keywords:** Polarized epithelial cells, Zebrafish, Disease model, Pathology, Epithelial tissues

## Abstract

Zebrafish (*Danio rerio*) have emerged as an influential model for studying human epithelial pathology, particularly because of their genetic similarity to humans and their unique physiological traits. This review explores the structural and functional homology between zebrafish and human epithelial tissues in organs, such as the gastrointestinal system, liver, and kidneys. Zebrafish possess significant cellular and functional homology with mammals, which facilitates the investigation of various diseases, including inflammatory bowel disease, nonalcoholic fatty liver disease, and polycystic kidney disease. The advantages of using zebrafish as a model organism include rapid external development, ease of genetic manipulation, and advanced imaging capabilities, allowing for the real-time observation of disease processes. However, limitations exist, particularly concerning the lack of organs in zebrafish and the potential for incomplete phenocopy of human conditions. Despite these challenges, ongoing research in adult zebrafish promises to enhance our understanding of the disease mechanisms and regenerative processes. By revealing the similarities and differences in epithelial cell function and disease pathways, this review highlights the value of zebrafish as a translational model for advancing our knowledge of human health and developing targeted therapies.

## Background

A comprehensive understanding of epithelial tissue diseases represents a crucial strategy for developing more effective treatments for human diseases. Animal models can uncover and verify various pathophysiological conditions to screen for effective therapies. Zebrafish (*Danio rerio*) has become an exceptional model over the last few years in different topics, such as development, diseases, organ function, vertebrate behavior, toxicology, and drug discovery research [1–6]. Recognizing the similarities in organ development between zebrafish and humans is crucial to reveal zebrafish’s ability to phenocopy epithelial tissue diseases.

The high level of conservation in the developmental physiology between humans and zebrafish has led to the use of zebrafish as a model for various health conditions. There is approximately 70% functional homology between zebrafish and human disease genes [7, 8]. Significant conservation has also been found between humans and zebrafish at the molecular level, regulating signal transduction and growth processes [9, 10]. Moreover, genome editing and transgenic and mutant fish production have made zebrafish a successful disease model. However, there are some limitations in utilizing zebrafish as models for diseases due to anatomical and physiological differences; zebrafish are missing some typical mammalian anatomical features like lungs, mammary glands, heart septation, synovial joints, or cancellous bone [11]. The zebrafish genome contains many duplicated genes. The original functions of the ancestral gene may be divided between two gene copies (a process known as sub-functionalization), or the gene copies may acquire entirely new functions (a phenomenon called neofunctionalization) [12]; this may require additional effort to verify functional roles. Additionally, some differences in tissue structure and gene function between zebrafish and humans may result in only partially phenocopied human disorders when zebrafish are used as a model [11, 13].

In different living organisms, polarized epithelial cells are vital components of various organs, such as the kidneys, gastrointestinal tract, and liver [14]. These cells have garnered the attention of researchers because of their vital functions, which underscore their pivotal roles in both health and disease processes [15].

Through a deeper understanding of the similarities and distinctions of zebrafish with human physiology, particularly in epithelial cell function and disease mechanisms, researchers can advance translational perceptions that support the development of targeted therapies for various human conditions.

## Main text

### Zebrafish and humans: a comparison of polarized epithelial cell structure and function across organs

#### Structure and development of the zebrafish gastrointestinal system

The digestive tract of zebrafish features a lengthy intestine that makes two bends within the abdominal cavity. The intestine is divided into three distinct regions: the intestinal bulb, mid-intestine, and posterior intestine. As the intestine progresses in a rostrocaudal direction, it folds to become shorter, with the posterior intestine consisting of a simple straight monolayer of simple columnar epithelial cells [16, 17]. The most prevalent intestinal epithelial cell types in zebrafish are goblet cells and columnar-shaped absorptive enterocytes.

In zebrafish, the term “anlagen” refers to early developmental structures that will eventually differentiate into specific organs or tissues. These anlagen are closely associated with the primitive gut tube, also known as the archenteron, which forms during gastrulation. The primitive gut tube serves as a precursor to the digestive tract and is essential for organizing the internal architecture of the embryo. The adjacency of these anlagen’s to the gut tube is critical for proper organ development, as interactions between adjacent tissues and signaling pathways govern cellular differentiation [18]. Key signaling molecules, such as Nodal and Bone Morphogenetic Protein (BMP), play significant roles in the specification of these anlagen, influencing the fate of cells that form the gut lining and associated organs, including the liver and pancreas [19]. By manipulating genes, researchers can gain insights into gut development and anlagen formation, providing findings that are often applicable to mammalian development [20].

**Fig. 1:**
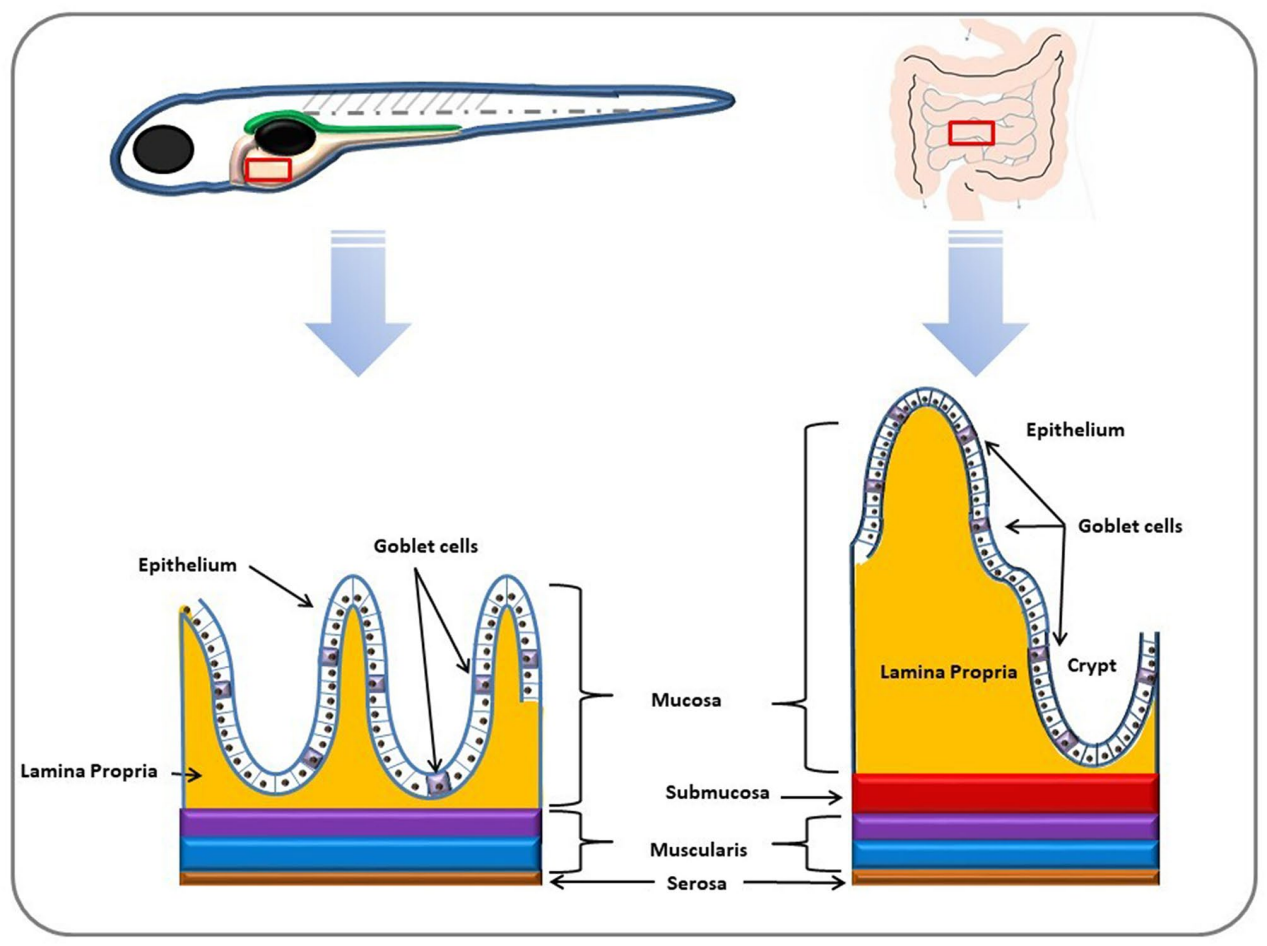
The zebrafish intestine has a simpler construction than that in mammals. Well-defined crypts and villi are absent in the zebrafish intestine. The zebrafish intestine has the same layers as in mammals, except for the submucosal layer, which is absent in zebrafish

Morphologically, zebrafish possess an intestinal bulb instead of a stomach, and their digestive system features a pointed channel, refers to its digestive system, which is structured as a tapered tube, divided into three distinct sections: intestinal bulb, midgut, and hindgut. The intestinal bulb functions as a food reservoir during embryonic and larval stages, facilitating the initial processing of ingested material. Following the intestinal bulb, the midgut is responsible for further digestion and nutrient absorption, whereas the hindgut plays a role in waste processing and absorption of water and ions. Notably, zebrafish lack gastric glands and genes associated with specific gastric functions, which differentiate their digestive system from that of mammals. Although the intestinal bulb is prominent in embryos and larvae, its enlargement is not observed in adult zebrafish, reflecting adaptation to their unique digestive physiology [17, 21–23] (Fig. 1) and (Table 1).

**Table 1:** A comparative overview of the anatomical and functional characteristics of zebrafish (*Danio rerio*) and human tissues within the gastrointestinal system, liver, and kidney

Feature	Zebrafish	Human
Gastrointestinal System		
Intestinal Structure	Three regions: intestinal bulb, mid-intestine, and posterior intestine	Intestine divided into duodenum, jejunum, ileum
Stomach	No stomach, intestinal bulb instead	Presence of stomach with gastric glands
Goblet Cells	Present	Present
Absorptive Enterocytes	Present	Present
Esophagus and Pharynx	Separated	Adjacent in the primal gut tube
Gastric Glands	Absent	Present
Peyer’s Patches	Absent	Present
Liver		
Lobes	Three distinct lobes	Typically divided into right and left lobes
Hepatocyte Arrangement	Not arranged in bilayered plates, no cord-like organization	Arranged in bilayered plates, cord-like organization
Bile Canaliculi	Intrahepatic bile ducts dispersed between hepatocytes	Bile canaliculi located between hepatocytes
Kupffer Cells	Absent	Present
Portal Triads	Not noticeable	Noticeable and well-defined
Kidney		
Structure	Pronephros in larvae, mesonephros in adults	Metanephros
Nephrons	Fewer and simpler nephrons	Complex and numerous nephrons
Glomeruli	Single glomerulus in larvae, multiple in adults	Multiple glomeruli
Tubules	Simple tubular structure	Complex tubular structure with distinct segments
Regenerative Capacity	High regenerative capacity	Limited regenerative capacity

#### Structure and development of the human gastrointestinal system

The human gastrointestinal (GI) tract is a complex organ system essential for digestion, nutrient absorption, and waste elimination. Anatomically, it is divided into several regions, including the esophagus, stomach, small intestine, and large intestine, each of which possesses specialized structures and functions. The esophagus connects the throat to the stomach, facilitating food movement via peristalsis. The stomach acts as a food reservoir, where it mixes with gastric juices to initiate the digestive process. The small intestine, which comprises the duodenum, jejunum, and ileum, is primarily responsible for nutrient absorption and is characterized by its extensive surface area due to the presence of villi and microvilli [24]. The large intestine is mainly involved in water absorption and fecal formation [25].

The epithelial lining of the GI tract consists of various cell types, each fulfilling a specific role. Absorptive enterocytes, which are columnar epithelial cells, are responsible for nutrient uptake and possess microvilli that enhance their absorptive capacity [26]. Goblet cells secrete mucus, providing lubrication and protection to the intestinal lining, thereby facilitating the smooth passage of food and preventing epithelial damage [27]. Enteroendocrine cells produce hormones that regulate digestive processes and influence gastric motility and insulin secretion [28]. Paneth cells, which are located in the small intestine, secrete antimicrobial peptides and enzymes that play a critical role in gut immunity and maintain the balance of the intestinal microbiome [29]. Additionally, the M cells found in Peyer’s patches are involved in the transport of antigens from the gut lumen to immune cells, thereby contributing to immune surveillance [30].

Anlagen in humans, which adjoins the primal gut tube during early embryonic development, is critical for the formation of various gastrointestinal structures. The anlagen develop from the primitive digestive tube, which forms through evagination, elongation, and dilatation by the fourth week of gestation. The primitive gut tube, which arises from the endoderm, is a key feature of the developing embryo, and its formation is marked by the emergence of a specific diverticula that gives rise to different organ systems [31, 32]. Intestinal stem cells within the crypts give rise to various epithelial cell types through proliferation and differentiation, regulated by signaling pathways such as Wnt, Notch, and Hedgehog [33]. These pathways are crucial for maintaining the balance between stem cell renewal and differentiation. Postnatally, the epithelial lining continues to undergo constant turnover and regeneration, influenced by interactions with the gut microbiome, which plays a vital role in shaping the immune landscape and the overall health of the GI tract [34].

## The differences in gastrointestinal system between zebrafish and humans

The gastrointestinal (GI) systems of zebrafish and humans exhibit several key differences in structure, function, and development. The following outlines the principal distinctions.

### Structural differences

#### Absence of stomach

Zebrafish possess a distinctive digestive system that markedly diverges from that of humans as they lack a true stomach. Instead, they feature a simple tubular structure comprising an intestinal bulb, mid-intestine, and posterior intestine, which collectively facilitates digestion and nutrient absorption. The intestinal bulb functions comparably to the stomach by assisting in initial digestion, while the mid-intestine is primarily responsible for nutrient absorption and the posterior intestine manages waste processing and excretion. This streamlined anatomical configuration renders zebrafish an advantageous model for investigating digestive processes and gut-microbiome interactions [21, 23, 35].

#### Intestinal structure

The human intestine possesses villi and crypts that enhance the surface area for absorption, whereas the intestines of zebrafish are characterized by irregular folds and absence of crypts. Zebrafish intestines are delineated into segments (S1 to S7), with S1-S4 exhibiting similarities to the mammalian small intestine, and S6-S7 displaying characteristics akin to the large intestine [23].

#### Cell types

Zebrafish are distinguished by unique intestinal cell types, particularly lysosome-rich enterocytes (LREs), which continue to be present in adulthood. In contrast, such cells are observed during the neonatal stage in mammals. This distinction emphasizes the evolutionary adaptations in nutrient absorption mechanisms across different species. LREs play a crucial role in enhancing dietary protein absorption and are integral to nutrient uptake throughout zebrafish life. The retention of these specialized cells enables zebrafish to sustain continuous nutrient absorption and efficiently adapt to dietary fluctuations, underscoring a significant divergence from mammalian physiology [36].

It is important to mention that zebrafish do not possess paneth cells, which are specialized secretory cells found in the intestines of many other vertebrates, including humans. Paneth cells play a crucial role in maintaining intestinal homeostasis and supporting intestinal stem cells by secreting antimicrobial peptides and growth factors. Zebrafish maintain intestinal homeostasis through various mechanisms, including the regulation of gut microbiota and epithelial cell regeneration [23, 37].

### Functional differences

#### Digestive enzymes

The types and activities of digestive enzymes vary between zebrafish and humans, reflecting their respective diets and processes. Zebrafish have adapted to a diet that may include microorganisms, whereas humans with a more varied diet require a broader spectrum of digestive enzymes [38].

For example, the main difference in lipid digestion lies in the presence and efficiency of lipase. Humans have multiple copies of the pancreatic lipase (PL) gene, an enzyme crucial for the proper metabolism of dietary fat. However, in zebrafish, this gene is completely absent, marking an important evolutionary difference in lipid metabolic pathways [39]. Another lipase, bile salt-activated lipase (BSAL), is present in different species but exhibits different characteristics. Zebrafish possess some BSAL, while humans rely on these enzymes, indicating differences in their reliance on BSAL for lipid digestion [39].

Protein digestion also demonstrates evolutionary variations. In humans, carboxypeptidase A, an important protease involved in breaking down proteins, is fully functional. In zebrafish, this enzyme is not completely developed due to mutations that impair pancreatic development [40]. This discrepancy highlights the evolutionary specialization of digestive enzymes in response to dietary and physiological needs.

Conversely, zebrafish exhibit a broader spectrum of sialidases, enzymes involved in glycan degradation, compared to humans. This expanded array suggests a differing enzymatic focus in their digestive processes, which may align with the unique dietary and environmental conditions zebrafish encounter [41, 42].

Humans and zebrafish share some digestive enzymes, but significant differences, such as the presence of pancreatic lipase and functional carboxypeptidase A in humans, reflect their distinct evolutionary adaptations to dietary and ecological demands. Zebrafish, with their expanded range of sialidase, demonstrate unique enzymatic specializations, emphasizing the complexity of evolutionary divergence in digestive biochemistry.

#### Microbiota interaction

The zebrafish model is now recognized as a vital means for exploring the complex interactions between the gut microbiome and the host organism. This model has significantly enhanced our understanding of how the microbiome influences host health, encompassing aspects such as immune functionality, metabolic processes, and vulnerability to inflammatory responses.

The gut microbiota exerts profound effects on various physiological processes critical to host health. Immune regulation is one of these primary roles, where microbial communities moderate host immune responses to maintain homeostasis and enhance resilience against infections [43]. Additionally, the microbiota contributes to metabolic functions by engaging in nutrient absorption and energy balance, such as carbohydrate, amino acid, and lipid metabolism, emphasizing its indispensable role in host metabolism [44]. Furthermore, intestinal epithelial dynamics are influenced by the microbiome through the promotion of epithelial cell differentiation and proliferation to preserve gut integrity [43].

Differences in gut microbiota are associated with various disease outcomes. For example, studies using zebrafish have shown that microbial composition can mediate exacerbations of infection by regulating host immune responses, particularly in the context of helminth parasites [45]. Additionally, microbial biomarkers and specific microbial enterotypes identified in zebrafish research suggest an association between hypertension and systemic conditions such as cardiovascular disease, highlighting the broader significance of infection to patient health [46].

Recent studies have highlighted several key findings: sub-chronic exposure to methyl induces dysbiosis, resulting in alterations to bacterial composition and intestinal morphology, which consequently leads to inflammation and issues related to nutrient metabolism; exposure to polystyrene nanoplastics is associated with structural changes and disruptions in microbiota, correlating with inflammatory responses and metabolic alterations; tick saliva has been demonstrated to modify the gut microbiota of zebrafish, potentially relating to allergic reactions in humans; and zebrafish have proven instrumental in gut-brain axis research, elucidating the influence of gut microbiota on neurodevelopment and behavior, with observations that may diverge from human responses [47–49].

### Developmental differences

#### Embryonic development

Zebrafish undergo rapid external development, and their intestines become functional shortly after hatching. In contrast, human intestinal development occurs internally and is marked by a more protracted period, with significant maturation during fetal development and continuing after birth [50].

#### Regeneration

Zebrafish possess remarkable regenerative capabilities, allowing for more effective recovery from intestinal injuries than humans. This regenerative capacity has been the focus of extensive research, particularly in elucidating tissue repair mechanisms [51, 52].

Zebrafish (*Danio rerio*) exhibits notable differences from humans, which enhances their utility as a research model for gastrointestinal physiology and diseases. Their digestive system is simpler than that of humans, facilitating the real-time observation of gastrointestinal processes owing to their transparent embryos. Furthermore, zebrafish possess exceptional regenerative capabilities, enabling investigation of recovery from gastrointestinal diseases. While they share structural and functional similarities with humans, such as comparable organ structures and a conserved gut-brain axis, their rapid development and genetic manipulability further facilitate the effective exploration of gastrointestinal mechanisms and disease models [53–55].

Other differences are also apparent between zebrafish and mammals; for instance, lymph glands and Peyer’s patches are absent in zebrafish [56]. But on the other hand, zebrafish thought to be an ideal model for studying host-microbe-immune interactions due to their transparent larvae, advanced genome editing technologies, and ability to be reared germ-freesuggesting recommendation that zebrafish could be a promising model studying gut development [21, 57].

## The similarities in gastrointestinal system between zebrafish and humans

The gastrointestinal (GI) systems of zebrafish and humans exhibit several notable similarities, rendering zebrafish a suitable model for investigating human digestive processes. Both species share fundamental structural and functional characteristics, which underscore their evolutionary conservation.

### Structural similarities

#### Cell types

Zebrafish and humans possess analogous cell types within their intestines, including absorptive enterocytes and goblet cells. These cell types play a critical role in nutrient absorption and mucus secretion. However, a notable distinction exists: zebrafish uniquely retain lysosome-rich enterocytes (LREs) into adulthood, whereas these cells are primarily present during the neonatal stage in humans [36, 58].

#### Intestinal segmentation

The intestines of both species are organized into distinct segments that fulfill specific functions. In zebrafish, the intestine is divided into seven segments (S1 to S7), with segments S1 to S4 resembling the mammalian small intestine, while segments S6 and S7 exhibit characteristics similar to the large intestine [23, 59]. This segmentation facilitates efficient absorption of nutrients and water, paralleling the functional organization observed in the human intestines.

The mucosal layer in both zebrafish and human intestines is essential for protection against pathogens and for facilitating digestion, primarily through the secretion of mucin by the goblet cells. Mucus plays a significant role in immune responses in zebrafish, with maternal mucus providing protection against pathogens and regulating inflammation [60]. In humans, the mucus layer serves as a barrier against foodborne pathogens and studies have indicated that it is crucial for preventing bacterial adhesion and invasion [61]. Furthermore, the development and maturation of goblet cells are influenced by microbiota, which is vital for maintaining the integrity of the mucus barrier [62]. The interplay between mucus and goblet cells highlights the significance of these components in both species.

### Functional similarities

#### Digestive enzymes

Zebrafish and humans synthesize a variety of digestive enzymes that facilitate the breakdown of macromolecules. Although the specific types and activities of these enzymes vary due to dietary adaptations, zebrafish primarily ingest microorganisms, whereas humans have a more diverse diet. The fundamental processes of digestion and nutrient absorption remain conserved [63] [64].

#### Immune response

The immune system of the zebrafish is characterized by a complex and highly efficient innate immunity necessary for survival in an aquatic environment. Zebrafish serve as valuable models for studying immune capacity due to their genetic similarities to humans, facilitating real-time visualization of immune processes. Our understanding of innate immunity, anti-inflammatory responses, and the influence of environmental factors on immune function has improved significantly.

Zebrafish primarily depend on their innate immune system, which plays an essential role in fighting infection and maintaining homeostasis. Its main components are neutrophils and macrophages-immune cells important in inflammation, anti-inflammation, and tissue regeneration. These cells mount rapid and dramatic responses to environmental challenges, demonstrating the flexibility of the zebrafish immune system [65].

The zebrafish model is particularly valuable in the study of innate immunity, especially in the early stages of development when a functional adaptive immune system is absent. This characteristic makes zebrafish ideal for understanding mechanisms of antiviral defense. Advanced techniques, such as CRISPR/Cas9 gene editing, have been used to explore specific genes and pathways involved in this response, providing important insights into host-pathogen interactions [66].

Both species exhibit analogous immune responses in their gastrointestinal tracts. The distribution of antimicrobial gene expression and leukocyte populations along the intestinal axis are relatively conserved, indicating a shared evolutionary strategy for maintaining gut health and responding to microbial challenges [23].

#### Developmental processes

Zebrafish undergo rapid external development, and their intestines become functional shortly after hatching. In contrast, human intestinal development occurs internally and is prolonged, with significant maturation occurring during fetal development and after birth. Despite these differences in developmental timing, the basic mechanisms of intestinal development, including the roles of signaling pathways and gene expression, demonstrate remarkable conservation between the two species [67].

These shared pathways and gene expression patterns underscore the evolutionary conservation of digestive organogenesis, even as species exhibit differences in developmental timing and complexity. The Bone Morphogenetic Protein (BMP) signaling pathway plays a fundamental role in the development of the enteric nervous system (ENS), which is essential for gut function. BMP signaling regulates the proliferation and differentiation of enteric neural progenitors, facilitating the colonization and functional maturation of the intestine [68]. Additionally, epigenetic modifications, such as those mediated by Wdr5 and histone trimethylation at lysine 4 (H3K4me3), are vital for digestive organogenesis. These modifications coordinate cell differentiation and proliferation, ensuring the proper morphogenesis of intestinal tissues [69].

Transcription factors are among the important players in intestinal development. Recent studies have identified 40 transcription factors with increased expression in the larval zebrafish gut, some of which are novel regulators of gut regionalization and motility [70] Furthermore, conserved signaling pathways, such as Wnt and Notch, along with epigenetic regulation-particularly through chromatin-modifying enzymes such as Ezh2-have been shown to maintain intestinal tissue integrity. Ezh2-mediated modifications facilitate chromatin stability and transcriptional regulation, underlining the importance of epigenetics in intestinal development [71].

The similarities between the gastrointestinal systems of zebrafish and humans underscore the utility of zebrafish as a model organism for biomedical research. Understanding these similarities not only enhances our knowledge of digestive physiology but also provides insight into potential therapeutic approaches for gastrointestinal diseases in humans.

## Studies on zebrafish gastrointestinal system

Transgenic zebrafish strains, such as the gutGFP line, have been developed in which green fluorescent protein (GFP) is specifically expressed solely within the endoderm and endoderm-derived organs from 22 h post-fertilization (hpf) onwards, extending through adulthood. The gutGFP strain has been used to study liver morphogenesis and development in zebrafish [72]. Another transgenic line, Tg [*nkx2.2a*: mEGFP], has also been used to explore the development of intestinal enteroendocrine cells [17]. Enhanced green fluorescent protein (EGFP) is induced by *nkx2.2a* in the brain, ventral neural tube, and developing pancreas. EGFP is expressed in cells that exhibit the characteristic morphological features of enteroendocrine cells [73].

Another transgenic line was produced by expressing GFP in the growing intestine, using the intestinal fatty acid-binding protein (*ifabp*) promoter. It has been shown that two GATA-type binding sites, one C/EBP binding site, and a 15-bp element in the I-FABP gene promoter participate in functionally conserved intestinal-specific gene expression between mammals and zebrafish [74] (Table 2).

**Table 2:** Transgenic Lines and mutants in zebrafish for Organ Research An overview of various transgenic lines and mutants in zebrafish, focusing on their applications in studying the kidney, liver, and gastrointestinal tract

Organ	Transgenic Line/Mutant	Description	Citation
Kidney	Tg(WT1:GFP)	Expresses GFP in podocytes, allowing visualization of kidney development and function.	[167]
Kidney	Tg(wt1b: EGFP)	Fluorescent visualization of proximal tubules and glomerulus; used to study the role of Wilms tumor protein (wt1) in kidney development and nephrogenesis.	[168]
Kidney	plce1 mutant	Knockdown leads to early onset nephrotic syndrome and end-stage kidney disease; involved in podocyte function and glomerular barrier integrity.	[169]
Kidney	fat1 mutant	Knockdown results in pronephric cyst development; important for glomerular function and migration of renal tubular cells.	[170]
Kidney	Tg (podocin: GFP)	Podocin-GFP zebrafish enable imaging and analysis of podocytes	[139]
Kidney	Tg(wt1b: GFP)	Used to visualize kidney structure and kidney cysts following wnt5a knockdown.	[164]
Liver	gutGFP line	Expresses GFP in endoderm-derived organs, aiding in liver morphogenesis studies.	[72]
Liver	Tg(lfabp: EGFP)	Used to study liver development and metabolism; expresses GFP in hepatocytes.	[55]
Liver	GFP under liver-specific promoters	Enables visualization of liver development and gene function investigation.	[55]
Liver	Tg(fabp10a: GFP)	Labels hepatocytes for liver function studies.	[171]
Liver	Tg(tp1:EGFP)	Labels cholangiocytes to study biliary function.	[172]
Liver	Tg(fabp10a: hCYP3A4-mCherry)	Humanized model for drug metabolism studies.	[173]
GI Tract	Tg(gutGFP)	Expresses GFP in the endoderm and gut, used for studying gastrointestinal development.	[72]
GI Tract	Tg(cdx1:GFP)	Marks intestinal progenitor cells, useful for studying intestinal development.	[174]
GI Tract	Tg [*nkx2.2a*: mEGFP]	Enteroendocrine cells are distinguished first at 52 hpf in the caudal region of the intestine	[17]
GI Tract	Monoclonal antibodies	Developed to study secretory and absorptive epithelial cell populations.	[75]
GI Tract	Morpholino knockdown technique	Investigated the role of krüppel-like factor 4 (Klf4) in intestinal cell proliferation and differentiation.	[76]
GI Tract	Tg(krt18:EGFP)	Labels intestinal epithelial cells for developmental studies.	[40]

Monoclonal antibodies were generated to facilitate the study of secretory and absorptive epithelial cell populations in the zebrafish intestine. Building on this work, Crosnier et al. investigated the role of Delta-Notch signaling in regulating diverse intestinal cell types. For instance, labeling with bromodeoxyuridine (BrdU) revealed a resemblance between the mechanisms governing gut epithelium renewal in zebrafish and their mammalian counterparts. Crosnier concluded that Delta-Notch signaling plays a crucial role in generating the appropriate combination of absorptive and secretory cells within the intestine. The critical role of Notch signaling in regulating the gut stem cell system highlights the usefulness of zebrafish as an important model organism for exploring the mechanisms that govern gut epithelial regeneration [75].

In conjunction with the morpholino knockdown technique, zebrafish were used to explore the role of Krüppel-like factor 4 (Klf4), which is essential for the final differentiation of goblet cells in the colon, as well as for intestinal cell proliferation and differentiation [76]. Various approaches have been used to investigate the development of the zebrafish digestive system, including light and fluorescence microscopy, histological staining, and specific antibodies that label cells, tissue compartments, and different transgenic lines. These tools help to visualize and analyze various components of the digestive system, including goblet cells, enteroendocrine cells, absorptive cells, and hepatocytes in zebrafish [77].

Intestinal epithelial cells (IECs) are essential for nutrient absorption and barrier function, with dysfunction linked to diseases such as inflammatory bowel disease and colorectal cancer. Additional studies have shown that many evolutionarily conserved regulatory regions, even without obvious sequence conservation, can drive conserved patterns of gene expression in the intestine, particularly when evaluated using fluorescent reporter assays in transparent zebrafish embryos. For instance, a highly conserved genomic region near the *hes1* gene initiates expression in a discrete population of IECs exhibiting active Notch signaling. Zebrafish models reveal evolutionary adaptations in intestinal anatomy, including at least five distinct segments: the anterior, middle, posterior intestines, and additional regions such as intestinal bulb and hindgut that show functional similarities to mammalian intestines. The conserved gene expression patterns in IECs across vertebrates emphasize the importance of zebrafish for studying IEC biology and its implications for understanding human gastrointestinal health and disease [78].

Zebrafish have emerged as a prominent vertebrate model system, with more than 40,000 research studies utilizing this powerful platform. The development of transgenic zebrafish lines expressing fluorescent proteins in specific cell types and tissues, including the hematopoietic system, urogenital system, digestive system, and even intracellular organelles, has revolutionized the real-time visualization and tracking of molecular, cellular, and organ-level processes. As genetic and imaging technologies continue to advance, transgenic fluorescent zebrafish are poised to enable unprecedented in vivo observation and manipulation of biological systems [79] (Table 3) and (Table 2).

**Table 3:** A summary of key findings from studies comparing zebrafish (*Danio rerio*) and human tissues in the gastrointestinal system, liver, and kidney

Study Area	Key Findings
Gastrointestinal System	
Morphological Development	Zebrafish intestine has three regions; no stomach present; intestinal bulb acts in its place.
Cell Types	Presence of goblet cells and absorptive enterocytes similar to humans.
Immune Structures	Lack of structures like Peyer’s patches found in humans.
Liver	
Liver Structure	Zebrafish liver has three lobes; different hepatocyte arrangement compared to humans.
Cellular Components	Absence of Kupffer cells in zebrafish; different organization of bile canaliculi.
Developmental Stages	Liver development studied using transgenic lines; occurs in two stages: budding and growth.
Kidney	
Kidney Structure	Zebrafish have pronephros in larvae and mesonephros in adults; humans have metanephros.
Nephron and Glomeruli	Zebrafish have fewer and simpler nephrons; single glomerulus in larvae, multiple in adults.
Regenerative Capacity	High regenerative capacity in zebrafish kidneys; limited in humans.
Experimental Techniques	
Genetic Tractability	High in zebrafish; ease of genetic modifications (e.g., CRISPR/Cas9, morpholinos).
Imaging Techniques	Advanced real-time visualization in zebrafish; allows detailed study of developmental processes.

In conclusion, the availability of zebrafish transgenic lines such as gutGFP and Tg[nkx2.2a: mEGFP] has critically advanced our understanding of embryonic development and physiology. These models have not only facilitated the study of specific cell types, such as intestinal endocrine and goblet cells but have also revealed the roles of important signaling pathways, including Delta-Notch and Kruppel-like factor 4, in fetal tissue metabolism and regeneration. Additionally, the use of monoclonal antibodies and advanced imaging techniques facilitates the visualization and analysis of complex phenomena in real time.

The identification of conserved regulatory regions that drive gene expression in the embryo highlights the evolutionary importance of zebrafish as a model organism. Their transparent embryos and the ability to express fluorescent proteins allow for unprecedented in vivo studies that can yield insights into human gastrointestinal health and disease. As research continues with these new tools, zebrafish will play an increasingly important role in uncovering transcriptional networks and regulatory pathways that shape vertebrate biology, contributing to advances in medical research and therapeutic strategies.

## Structure and development of the zebrafish liver

Hepatocytes are the primary functional cells of the liver. They are responsible for executing most of the essential functions of the organ, including bile production, blood detoxification, plasma protein synthesis, and the formation of coagulation factors. The liver of zebrafish is anatomically distinct and consists of three lobes that extend longitudinally along the intestinal tract. Despite these differences, the zebrafish liver shares several key functional similarities with the human liver, including metabolic and synthetic activities [80, 81].

The zebrafish liver typically begins to develop around 24 h ‘post-fertilization (hpf) and reaches full maturity by approximately 5 days’ post-fertilization (dpf) [82]. This rapid organogenesis is facilitated by the external fertilization of zebrafish and its embryo transparency, allowing for real-time observation of developmental processes.

The liver originates from the anterior endoderm, where a series of transcription factors and signaling pathways orchestrate the differentiation of hepatoblasts, liver precursor cells. Key transcription factors involved in this process include members of the FoxA, Gata, and Hnf families, which are also essential for mammalian liver development [82, 83]. During hepatogenesis, the liver bud forms as hepatoblasts proliferate and migrate to the surrounding mesenchyme. This process is tightly regulated by various signaling pathways, notably fibroblast growth factor (FGF) and bone morphogenetic protein (BMP) signaling, which ensures the proper specification and maturation of hepatoblasts into functional hepatocytes.

The development of hepatic vasculature is also crucial, as it co-develops with hepatoblasts and supports liver function and hematopoiesis [83]. Transgenic zebrafish lines, such as those expressing green fluorescent protein (GFP) under liver-specific promoters, have enabled researchers to visualize liver development and to investigate the roles of specific genes in this process [55] (Table 2).

## Structure and development of the human liver

The human liver is an essential organ characterized by a complex structure and plays a critical role in metabolic processes, detoxification, and bile production. Anatomically, the liver is divided into two principal lobes, which are further subdivided into lobules—small functional units composed of hepatocytes organized around a central vein. These lobules are structurally designed to facilitate efficient blood flow from the hepatic portal vein and hepatic artery, thereby allowing optimal nutrient processing and detoxification. The distinctive architecture of the liver is characterized by a rich supply of blood vessels, bile ducts, and supportive extracellular matrix, reflecting its multifaceted functions [84].

Developmentally, the liver originates from the endodermal germ layer in the early stages of embryogenesis. It initiates as a hepatic diverticulum that emerges from the foregut around the third week of gestation. This diverticulum undergoes a series of complex morphogenetic processes including branching and differentiation to form a mature liver [85]. Hepatoblasts, the progenitor cells of the liver, undergo proliferation and differentiation into hepatocytes and cholangiocytes (bile duct cells) under the influence of various signaling pathways including the Wnt and Notch pathways [86]. In the first trimester, the liver is capable of performing several essential functions, including hematopoiesis, and continues to develop both functionally and structurally throughout fetal life and early childhood [87]. A comprehensive understanding of the intricate structure and developmental processes of the liver is critical for gaining insight into liver diseases and regenerative medicine.

**Fig. 2:**
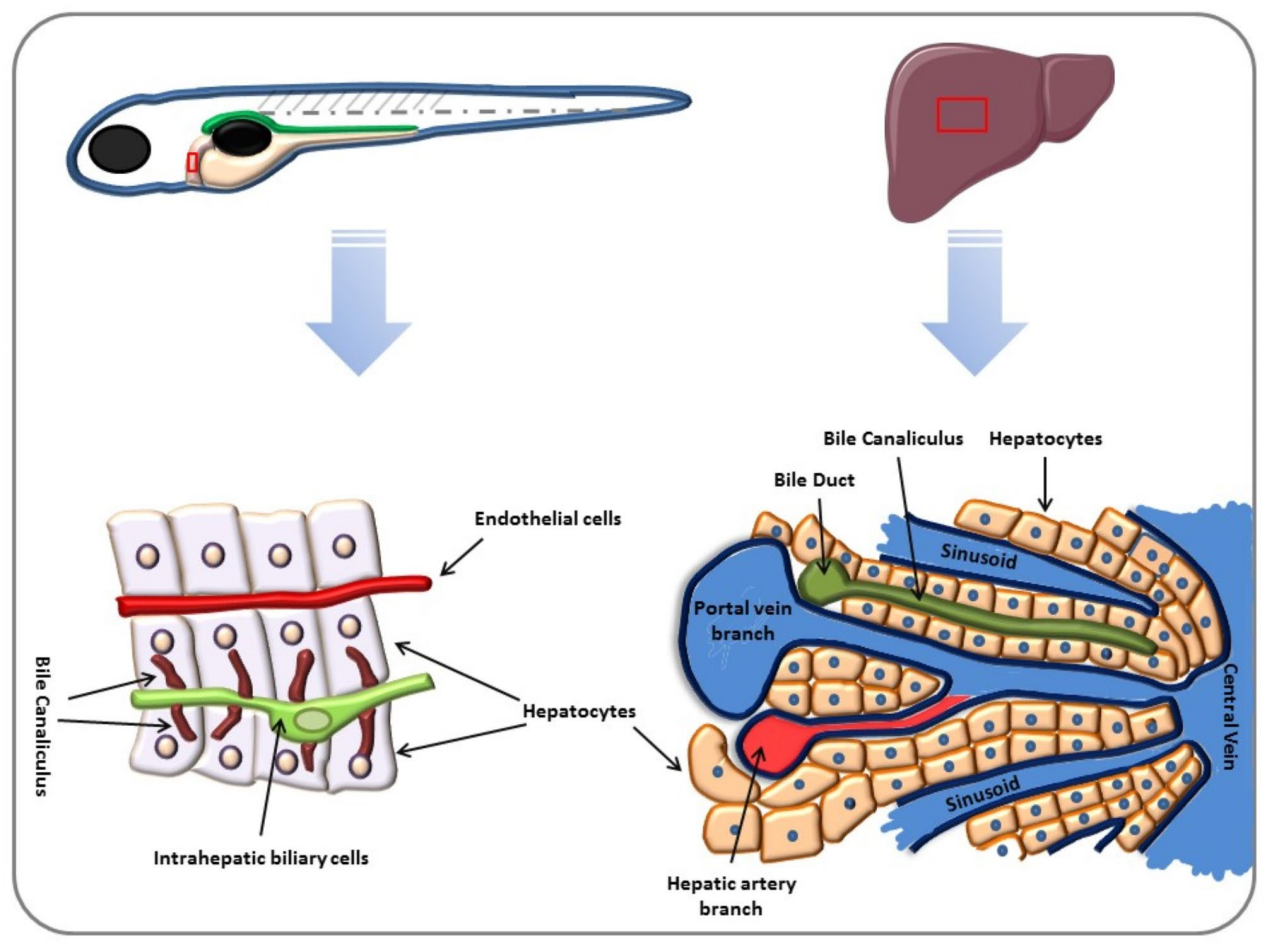
Structural similarity between zebrafish and mammalian liver. They both contain polarized hepatocytes maintained by biliary epithelial cells and the bile canaliculus. On the other hand, intrahepatic biliary ductile in zebrafish was positioned on the apical side of the hepatocytes. In addition, hepatocytes disjoin the intrahepatic bile ducts from the blood vessels, whereas in mammals, bile canaliculi are situated between hepatocytes. In zebrafish, bile canaliculi are the invagination of the apical membrane of hepatocytes which is associated with the biliary ductules

## The differences in the liver between Zebrafish and humans

### Structural organization

The histological organization of the zebrafish liver differs significantly from that of mammals. In mammals, hepatocytes are arranged in bilayered plates with bile canaliculi situated between them. In contrast, hepatocytes in zebrafish lack this cord-like organization, and intrahepatic bile ducts, portal veins, and hepatic arteries are dispersed randomly within the hepatic parenchyma [40, 88]. (Fig. 2). Notably, Kupffer cells, which play a crucial role in immune responses in mammals, are absent in the zebrafish liver, and the portal triads are not distinctly formed [89].

The human liver is organized into distinct hepatic lobules, which represent the smallest functional units. Each lobule comprises plates of hepatocytes arranged around a central vein, facilitating the efficient flow of blood and bile. In contrast, the zebrafish liver consists of three contiguous lobes (two lateral and one ventral) that lack a clear lobular structure, complicating the understanding of blood flow dynamics within the liver [55, 90].

### Cellular architecture

In humans, hepatocytes are arranged in radial plates that extend outward from the central vein, with sinusoidal spaces enabling the exchange of substances between blood and liver cells. Conversely, zebrafish hepatocytes are organized in more tubular structures, which are less distinct than the lobular architecture observed in mammals. This difference affects metabolic functions and the overall efficiency of hepatic processes [90].

### Immune cell composition

Humans possess specialized immune cells known as Kupffer cells, which are resident macrophages located within the liver sinusoids. These cells play a crucial role in immune surveillance and the removal of pathogens. Zebrafish, however, do not have Kupffer cells; instead, they harbor a different population of immune cells that perform similar functions but may vary in their mechanisms and efficiency [91, 92].

### Regenerative capacity

Zebrafish exhibit remarkable regenerative abilities, allowing for efficient regeneration of liver tissue following partial hepatectomy or injury, a process that occurs more effectively than in humans. This regenerative capacity is characterized by the proliferation of hepatocytes and the mobilization of progenitor cells, a mechanism that is still under investigation in zebrafish. In contrast, while liver regeneration occurs in humans, it is frequently accompanied by fibrosis and scarring, which limits functional recovery [93].

### Biochemical pathways

The metabolic pathways in the liver can differ significantly between zebrafish and humans, influencing how each species processes nutrients and drugs. For instance, zebrafish exhibit distinct lipid metabolic pathways, that enable them to utilize dietary fatty acids more efficiently than humans. This adaptation reflects the aquatic environment of zebrafish, where lipid sources diverge from those found in terrestrial diets [94]. Furthermore, detoxification processes in zebrafish involve specific enzymatic pathways, including cytochrome P450 enzymes, which facilitate the metabolism of various xenobiotics and environmental toxins. These adaptations are essential for survival in habitats where exposure to pollutants is prevalent [95]. Research has indicated that zebrafish metabolize certain xenobiotics, such as pharmaceuticals and environmental chemicals, in a manner distinct from humans, exhibiting varying rates of drug metabolism and clearance. Such metabolic variability is critical for researchers utilizing zebrafish as a model organism for studying drug effects and toxicity [96]. Overall, an understanding of these differences in metabolic pathways enhances the utility of zebrafish in biomedical research and underscores the significance of species-specific responses in pharmacology and toxicology.

## Similarities between the liver of zebrafish and humans

### Hepatic functionality

Both zebrafish and human livers perform critical metabolic functions that are essential for maintaining homeostasis and overall health. These functions include bile secretion, lipid and glycogen storage, detoxification of harmful substances, and amino acid metabolism. Bile produced by hepatocytes aids in fat digestion and absorption, with zebrafish exhibiting a unique biliary network. Both species store lipids and glycogen in the liver, ensuring energy availability during periods of metabolic demand. The detoxification processes, primarily mediated by cytochrome P450 enzymes, enable both species to effectively metabolize xenobiotics and environmental toxins. Additionally, the liver regulates amino acid levels, facilitating protein synthesis and various metabolic pathways in both zebrafish and humans [96–98].

### Cell type conservation

Major cell types in the liver, including hepatocytes, biliary epithelial cells (BECs), and hepatic stellate cells (HSCs), are conserved between zebrafish and humans, reflecting significant functional similarities. Hepatocytes, the predominant cell type, are responsible for most metabolic functions, such as protein synthesis, lipid and carbohydrate metabolism, detoxification, and bile production. BECs are critical for bile secretion, with zebrafish exhibiting a unique biliary architecture that facilitates bile transport within their aquatic environment. HSCs serve dual roles by storing vitamin A in a healthy liver and promoting fibrosis upon injury through the production of extracellular matrix components. This conservation of liver cell types underscores their evolutionary significance and provides a unique framework for studying liver biology and disease mechanisms in model organisms such as zebrafish [55, 99, 100].

### Genetic similarity

There exists a significant degree of conservation in liver-associated genes between zebrafish and humans, with approximately 70% of human genes possessing at least one orthologue in zebrafish. This genetic similarity is particularly advantageous for translational research in liver diseases, as it permits researchers to utilize zebrafish as a model organism for investigating various aspects of liver development, function, and pathology. The conserved genetic framework facilitates the exploration of mechanisms underlying liver diseases and the evaluation of potential therapeutic interventions within a system that shares fundamental biological processes with humans. Consequently, zebrafish have emerged as an invaluable tool in enhancing our understanding of liver disorders and in the development of novel treatment strategies [55, 83].

Recent studies utilizing single-cell RNA sequencing (scRNA-seq) have shown that zebrafish and human liver cells possess highly conserved transcriptional profiles, particularly in hepatic stellate cells (HSCs), which are crucial in the development of liver fibrosis [101]. The analysis indicates that zebrafish hepatic cell types, especially HSCs, express conserved marker genes, such as Subfamily Member 11 (colec11) and Neuropilin 1, which are also present in human liver cells. This genetic conservation highlights the utility of zebrafish as a model organism for investigating liver diseases and exploring the mechanisms underlying liver fibrosis [102].

### Response to injury

Both zebrafish and humans exhibit analogous cellular responses to liver injury, characterized by inflammation and the activation of tissue repair mechanisms. This process involves a complex interplay among various immune cells and molecular signals that promote recovery. Key immune components, such as macrophages, transition from a pro-inflammatory state to a reparative phase in both species, contributing to the restoration of homeostasis [103]. Furthermore, other immune cells, including neutrophils, T cells, and natural killer cells, infiltrate the liver during the injury and recovery phases, demonstrating distinct temporal patterns [104].

Molecular signaling pathways also play a critical role in these processes. Damage-associated molecular patterns (DAMPs), such as HMGB1 and CIRP, are vital for initiating immune responses, highlighting the significance of molecular interactions in liver injury [105]. Zebrafish are particularly important for investigating liver diseases due to their remarkable regenerative capabilities [55]. While the similarities in immune responses offer promising opportunities for therapeutic understanding, the complexity of human liver diseases may necessitate tailored approaches that extend beyond the modeling capabilities of zebrafish.

### Bile production

Both zebrafish and humans produce bile, which is essential for the emulsification and absorption of dietary fats. The mechanisms regulating bile secretion and composition are conserved between the two species, rendering zebrafish fit for studying bile-related disorders. In zebrafish, bile is synthesized by hepatocytes and secreted into the bile ducts, analogous to the process in humans [106, 107]. This conservation facilitates the investigation of the underlying mechanisms of bile production and its role in lipid metabolism, which may yield to human health and disease.

The ability to manipulate gene expression through techniques such as morpholino knockdown and CRISPR/Cas9 genome editing has enabled researchers to dissect the molecular pathways involved in liver development and disease [55, 83]. For example, the role of krüppel-like factor 4 (Klf4) in intestinal cell differentiation and proliferation has been explored, emphasizing the conservation of developmental mechanisms between zebrafish and mammals [76].

The liver of zebrafish and humans exhibits significant differences in structure, immune composition, and regenerative capacity; however, it shares many fundamental similarities in function, cell types, and genetic makeup. These revelations not only enhance our understanding of liver biology but also underscore the potential of zebrafish as a model organism in biomedical research, particularly concerning liver diseases and regenerative medicine. By leveraging these similarities and differences, researchers can more effectively investigate liver function, disease mechanisms, and potential therapeutic interventions.

## Studies on zebrafish liver

Many studies have been accomplished to discover the mechanisms that regulate liver morphogenesis and development. Despite extensive studies on the liver, particularly the hepatocytes, there is still much to be explored. Studying liver development in mammals is challenging and has some complications. It is not easy to learn mammalian liver development via a direct genetic approach because its embryonic development occurs inside the uterine [108]. Therefore, zebrafish was the choice for studying liver development processes.

In a study of liver development, Field et al. (2003) leveraged the optical transparency of the zebrafish model in combination with the gutGFP transgenic line to elucidate two distinct stages of liver morphogenesis: budding and growth. The budding phase commenced first, marked by the aggregation of hepatocytes, which occurred after 24 h post-fertilization (hpf) and culminated in the development of the hepatic duct by 50 hpf. Importantly, they found that the directionality of this budding process was independent of the concurrent looping of the intestine. The subsequent growth phase was characterized by observable changes in the liver’s shape and size. Furthermore, the study revealed that zebrafish endothelial cells are not essential for the initial budding of the liver [72] (Table 2).

Zebrafish have emerged as a valued model organism for investigating the initiation and development of the liver during embryogenesis. These studies have focused on elucidating the formation and maturation of the liver bud, the differentiation of hepatocytes and bile duct cells, as well as the role of mesodermal signals in the regulation of liver development [109].

The role of the hepatotrophic growth factor known as the ‘augmenter of liver regeneration’ (ALR) was investigated in the context of liver formation using the zebrafish model. This was accomplished through a combination of knockdown and overexpression techniques. Specifically, the knockdown of the *alr* gene, achieved via morpholino antisense oligonucleotide (MO) technology, was found to inhibit liver growth. Conversely, the overexpression of *alr* was observed to stimulate liver growth in zebrafish [110].

Research on biliary diseases, such as biliary atresia, has particularly benefited from leveraging the developmental biology knowledge gained from zebrafish studies. Beyond the developmental origins of disease, zebrafish are also being used to investigate other common liver pathologies, including diabetes, cancer, fibrosis, and toxin-induced injury, highlighting the broad applicability of this model system [111].

Zebrafish have proven to be an interesting model for studying a diverse array of liver diseases, including drug-induced liver injury, hepatocellular carcinoma, nonalcoholic fatty liver disease, and cholestasis [55, 112, 113]. However, it is important to recognize that there are several significant limitations to consider when utilizing zebrafish as a model for human liver diseases.

One significant limitation in applying zebrafish as a model for human liver diseases is that the zebrafish immune system, particularly the adaptive immune system, is not fully developed, especially during the larval stage [114, 115]. This can pose challenges in accurately recreating the human immune responses and inflammatory components associated with various liver diseases. Additionally, measuring key liver enzymes like Alanine Aminotransferase (ALT) and Aspartate Aminotransferase (AST) in zebrafish remains technically difficult due to the small blood volumes that can be collected without causing significant stress or injury to the animal [116].

Zebrafish demonstrated themselves as a beneficial model for investigating hepatocellular carcinoma (HCC) due to their similar liver function and development to humans, as well as the advantages of their genetic tractability, transparency, and low-cost housing, enabling chemical screens, live imaging, and dissection of genetic and biochemical modulators of liver biology. While zebrafish HCC models have limitations like the lack of clear liver zonation and challenges with antibody-based assays, they can significantly contribute to the HCC drug discovery pipeline, particularly through in vivo chemical screens that identify potentially efficacious compounds, which may not require full validation in traditional mammalian models Given the well-documented challenges in translating findings from murine studies to successful clinical outcomes, the limitations of the zebrafish model for liver disease research become even more critical to consider [117].

Another limitation is that there are physiological differences between zebrafish and human hepatocytes, including variations in metabolic pathways, drug metabolism, and detoxification processes. Furthermore, the fundamental anatomical differences between the zebrafish and human liver, such as the distinct lobular organization, vasculature, and biliary architecture, can significantly impact the localization and progression of disease processes being modeled. Careful consideration of these species-specific differences is crucial when interpreting findings from zebrafish liver disease models [55] (Table 3) and (Table 2).

## Structure and development of the zebrafish kidney

The zebrafish kidney, particularly the pronephros, is an exemplary model for investigating kidney structure, cellular composition, and developmental processes. The pronephros represent the initial stage of kidney develop development, originating from the intermediate mesoderm during gastrulation, influenced by morphogens such as Bone Morphogenetic Proteins (BMPs) and Nodal signaling [118, 119]. Structurally, the pronephros comprises specialized epithelial cells organized into segments that include glomeruli and tubules, which are essential for filtration and solute reabsorption [120, 121]. Each nephron segment is composed of distinct cell types, including glomerular progenitors, podocytes, and multi-ciliated cells, which facilitate the movement of filtrate and enhance overall kidney function [121].

The development of the pronephros involves critical signaling pathways, such as retinoic acid and Notch, which are indispensable for regulating cell fate and segment formation [122, 123]. These pathways coordinate the differentiation of nephron progenitor cells and the establishment of the kidney’s functional architecture. The pronephros develops rapidly during initial embryonic stages, typically forming by 24 h post-fertilization (hpf) and becoming functional by approximately 48 hpf, consisting of only two nephrons responsible for early excretory functions [121].

After the development of the pronephros, the mesonephros begins to form around 12–14 days’ post-fertilization (dpf) and serves as the primary kidney during the larval stage. The mesonephros is more intricate, containing hundreds of interconnected nephrons capable of executing advanced renal functions [119, 124]. Unlike mammals, zebrafish do not develop a metanephros; instead, the adult kidney, which is derived from mesonephric tissue, remains functional throughout the fish’s life, allowing for continuous nephron addition and regeneration [122, 125]. This remarkable regenerative capability represents a significant area of research, offering more knowledge about potential therapeutic approaches for kidney diseases in humans.

Kidney development in zebrafish necessitates the precise coordination of proteins, signaling molecules, and gene expression programs, as disruptions in these processes can result in severe renal pathologies [126]. Although the complexity of mammalian kidneys poses challenges for experimental studies, the simplified structure of the zebrafish pronephros provides a suitable alternative model system. Despite the differences in overall kidney architecture, the zebrafish pronephros exhibits key features similar to those of the mammalian metanephros, particularly in the highly conserved podocytes. This conservation underscores the potential of zebrafish as a model organism for elucidating kidney development and function, as well as for investigating the mechanisms underlying renal diseases.

## Structure and development of the human kidney

The human kidney undergoes a complex process of structural and development changes, commencing approximately 22 days’ post-fertilization and concluding between the 34th and 36th week of gestation. This developmental progression involves three sequential kidney forms: the pronephros, mesonephros, and metanephros, with the metanephros being essential for the formation of the functional adult kidney. Nephrogenesis begins with the emergence of the ureteric bud, which is crucial for the branching morphogenesis that leads to the formation of glomeruli by approximately 8 to 9 weeks of gestation [127].

The cellular dynamics during kidney development are characterized by the differentiation of nephron progenitor cells, which give rise to various components of the nephron, including glomeruli, proximal tubules, the loop of Henle, distal tubules, and collecting ducts. These structures are vital for the kidney’s filtration and regulatory functions [128]. Recent studies have identified distinct stages in nephron development, including a newly characterized W-shaped body stage, as well as various sub-stages of the capillary loop, which are critical for understanding nephron maturation and functionality [129].

Furthermore, comparative analyses of kidney development between humans and zebrafish have revealed both conserved and divergent features. These findings emphasize the importance of species-specific research to elucidate the mechanisms underlying nephrogenesis and kidney function [130]. Understanding these developmental processes is essential, as they enhance our knowledge of normal kidney function but also inform potential therapeutic strategies for congenital kidney disorders and other renal pathologies.

## Differences between the kidney of zebrafish and humans

Zebrafish and human kidneys exhibit significant differences in cell type, structure, and development, despite certain similarities that render zebrafish a model for renal research. The zebrafish kidney, primarily composed of the pronephros, is structurally simpler than the human kidney, which is characterized by a complex organization into distinct regions such as the cortex and medulla. This complexity in humans facilitates advanced filtration and waste excretion processes, which are more refined compared to the simpler filtration mechanisms present in zebrafish [124, 131].

**Fig. 3:**
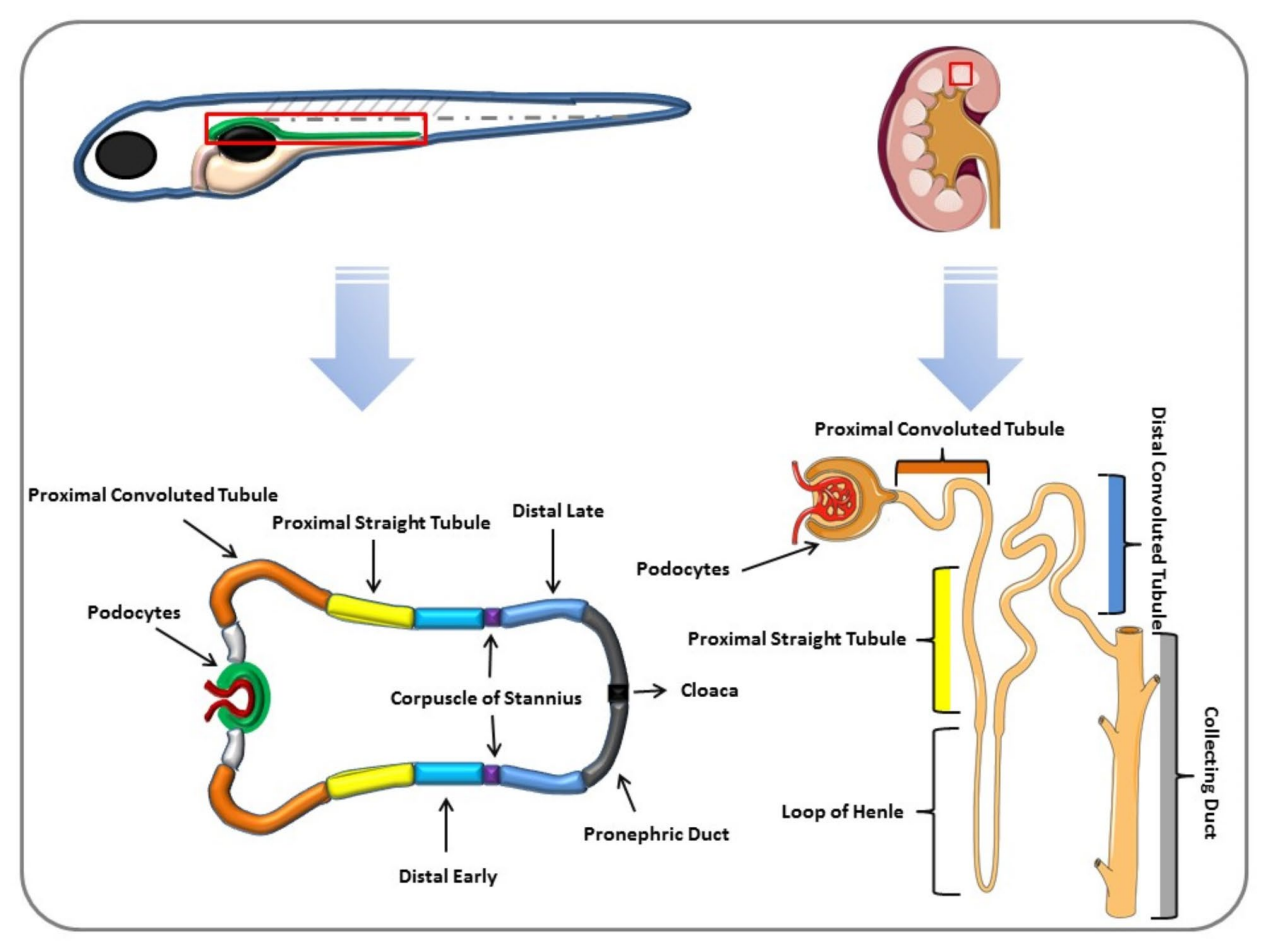
A high degree of similarity is noticed between the nephron segmentation patterns of both zebrafish and human. Human nephron contains podocytes, proximal convoluted tubule, proximal straight tubule, distal convoluted tubule, a loop of Henle and collecting duct. While zebrafish pronephros contains podocytes, proximal convoluted tubule, proximal straight tubule, distal early, corpuscle of stannius, distal late, and pronephric duct but there is no loop of Henle

In terms of cellular composition, both zebrafish and human kidneys contain nephron progenitor cells that differentiate into various nephron components. However, the zebrafish pronephros lacks the specialized structures found in human nephrons, such as glomeruli and the extensive tubular system that supports intricate regulatory functions [131]. Furthermore, zebrafish kidneys exhibit a high degree of genetic conservation, with 81 out of 82 human cystic kidney disease genes having zebrafish homologs, indicating a strong genetic similarity that can be leveraged for disease modeling. However, zebrafish also display sexual dimorphism in gene expression, which is less pronounced in humans, potentially influencing the outcomes of genetic studies [132] (Fig. 3).

Developmentally, zebrafish kidneys form rapidly, with nephrogenesis occurring within the first 24 h post-fertilization, while human kidney development spans several weeks, beginning around the 22nd day of gestation and continuing until the 34th to 36th week [133]. This rapid development in zebrafish allows for real-time observation of kidney formation and function, making them an effective model for nephrotoxicity studies and other renal research. However, the simpler anatomy and physiology of zebrafish may limit the direct applicability of findings to human kidney function and disease mechanisms, highlighting the necessity of employing multiple models in biomedical research to gain a comprehensive understanding of renal biology.

## Similarities between the kidney of zebrafish and humans

Despite the differences in overall kidney development, kidneys of zebrafish exhibit several structural similarities with those of mammals. Both zebrafish and human kidneys share multiple similarities in cell types, structures, and developmental processes, rendering zebrafish an interesting model for study of renal biology and associated diseases. Both species display a conserved nephron structure, which serves as the functional unit of the kidney responsible for filtration and waste excretion. The nephron in both zebrafish and humans consists of analogous components, including glomeruli, proximal tubules, and distal tubules, which perform comparable functions in fluid and electrolyte balance [124, 134].

At the cellular level, the kidneys of zebrafish and humans contain homologous cell types, such as podocytes and tubular epithelial cells, which play crucial roles in maintaining renal function. The molecular pathways governing nephron development are also highly conserved between the two species, with key signaling pathways, such as Notch and Wnt, involved in nephrogenesis in both zebrafish and humans. This genetic conservation is evident, as studies have indicated that a significant number of genes associated with human kidney diseases possess zebrafish counterparts, thereby facilitating the use of zebrafish in modeling human renal diseases [131, 134].

Developmentally, both zebrafish and human kidneys undergo a series of well-defined stages during nephrogenesis. In zebrafish, nephron formation commences shortly after fertilization, allowing for rapid observation of kidney development, while in humans, nephrogenesis unfolds over several weeks during embryonic development. Despite the differences in timing, the fundamental processes of nephron formation, including the differentiation of nephron progenitor cells and the establishment of the renal vasculature, exhibit remarkable similarity [127, 135]. Both species utilize comparable signaling pathways, including Wnt and BMP, which are crucial for nephron progenitor maintenance and differentiation [136]. These shared characteristics underscore the utility of zebrafish as a model organism for investigating kidney development and disease mechanisms relevant to humans.

## Studies on the zebrafish kidney

Different mutations have been induced in the zebrafish pronephros to assess their influence on pronephros growth and function. Fifteen recessive mutations were identified that interfere with pronephros development. In all these mutations, clear cysts were observed in the pronephric tubule at the 2–2.5 dpf stage. Additionally, a critical consequence was observed concerning a basolateral membrane protein expressed in the epithelial cells of the pronephric duct. This finding elucidates the similarities in pronephric kidney formation across all vertebrates [137].

Researchers have investigated the anatomical and physiological development of the nephron in zebrafish, with the goal of characterizing the mechanisms that regulate nephron segmentation. Subsequently, numerous studies have been conducted utilizing the zebrafish model to unravel the complexities of nephron segmentation mechanisms. Investigations into embryonic kidney development have revealed similarities in the segmentation of pronephric nephrons between zebrafish and mammals. This suggests the potential of using the zebrafish model to further comprehend the fundamental processes underlying nephron development and segmentation, which could have implications for elucidating the pathogenesis of human kidney disorders.

These findings illustrate the efficacy of zebrafish as a model organism to investigate the conserved developmental pathways governing nephron segmentation in both the lower vertebrates and mammals [138].

High conservation has also been identified between the zebrafish glomerulus and podocytes as compared to those in mammals leading to its employment as a model for glomerular disease model [137]. Moreover, the ultrastructural characterization of a podocyte-specific transgenic line, Tg (podocin: GFP), has demonstrated a high degree of similarity to podocytes in mammalian metanephric kidneys [139] (Table 2).

Kidney development requires precise coordination of proteins, signaling molecules, and gene expression programs. Disruptions in this intricate process can have severe consequences. The complexity of the mammalian kidney presents challenges for experimental studies. However, the simplified and genetically tractable zebrafish pronephros offers a valuable alternative model system. While there are differences in overall kidney structure between zebrafish and mammals, the zebrafish pronephros shares key similarities with the mammalian metanephros, particularly in the highly conserved podocytes. This has enabled experimental studies in zebrafish to provide important clarity into the fundamental mechanisms of podocyte ontogeny and development. As new methods and tools continue to be developed, the zebrafish model will remain an influential tool for the nephrology community to advance research in areas such as renal organoid technology and the discovery of new therapeutic strategies [140] (Table 3).

Zebrafish are increasingly recognized as a powerful model for studying kidney development due to their genetic tractability and the simplicity of their renal structures. The zebrafish kidney develops through distinct stages, beginning with the pronephros, which is the first functional kidney structure. This organ forms rapidly during early embryonic development, typically becoming functional by 48 h post-fertilization (hpf) and consists of two nephrons [121, 124].

Various mutations have been induced in zebrafish to assess their impact on pronephros growth and function. One study identified fifteen recessive mutations that disrupt pronephros development, leading to the formation of cysts in the pronephric tubules at 2 to 2.5 dpf. These mutations also affect a basolateral membrane protein expressed in the epithelial cells of the pronephric duct, highlighting the conserved mechanisms of pronephric kidney formation across vertebrates [122, 137].

Research has focused on the anatomical and physiological development of nephrons in zebrafish to elucidate the mechanisms regulating nephron segmentation. Studies have shown that the segmentation of pronephric nephrons in zebrafish exhibits significant similarities to that in mammals, suggesting that knowledge gained from zebrafish can enhance our understanding of human renal diseases. For instance, the conservation of nephron segment composition across species underscores the evolutionary significance of these developmental pathways.

High conservation has been observed between zebrafish glomeruli and podocytes in comparison to those found in mammals. The ultrastructural characterization of podocyte-specific transgenic lines, such as Tg (podocin: GFP), has revealed that zebrafish podocytes exhibit remarkable similarities to those present in mammalian metanephric kidneys [139]. This correspondence facilitates the investigations of podocyte development and functionality within a simplified model, thereby providing insights that may be applicable to human kidney disorders. See (Table 2).

As new methodologies and tools continue to emerge, the zebrafish model is anticipated to play a pivotal role in advancing nephrology research. This includes applications in renal organoid technology and the discovery of novel therapeutic strategies for kidney diseases [140]. The capacity to manipulate zebrafish genetics and observe developmental processes in real time renders this model particularly advantageous for understanding the fundamental mechanisms underlying nephron development and the pathogenesis of renal disorders.

## Zebrafish as a model for epithelial tissue diseases

### Gastrointestinal disease

Colitis, a significant form of inflammatory bowel disease (IBD), is characterized by chronic inflammation of the gastrointestinal tract, leading to debilitating symptoms and long-term health complications. The complexity of IBD pathogenesis, which involves genetic, environmental, and immunological factors, necessitates robust animal models for research. Numerous studies have emphasized the utility of adult zebrafish (*Danio rerio*) as a model organism for investigating the mechanisms underlying colitis and other forms of IBD [141].

While the adult zebrafish model offers considerable advantages, such as genetic manipulability and rapid development, it also presents limitations. The lack of transparency in adult zebrafish constrains detailed imaging of internal structures, making it challenging to visualize disease progression and cellular interactions. However, this limitation can be mitigated by using transgenic zebrafish lines, such as the Casper line, which maintain transparency into adulthood. This characteristic enable researcher to perform high-resolution imaging and track cellular dynamics in real time, thereby enhancing the understanding of disease mechanisms.

The transformative role of transgenic fluorescent zebrafish lines has been underscored in biomedical research since their introduction in 1981, emphasizing their capacity to express fluorescent proteins in specific cells and tissues for real-time visualization of biological processes. These zebrafish lines have significantly advanced studies in various areas, including signal transduction—where pathways such as BMP, Wnt, and FGF are explored—and the gastrointestinal system, where researchers can track cellular developments. The transparency of zebrafish during early development facilitates detailed observation, making them indispensable for unraveling complex biological systems and driving innovations in genetics and disease modeling. Overall, these transgenic lines are essential tools for contemporary biomedical research, offering perspectives on potential therapeutic applications [79].

One study has demonstrated that adult zebrafish can effectively model intestinal inflammation. For instance, a study developed a soy-dependent model of intestinal inflammation in adult zebrafish, wherein a diet rich in soybean meal induced significant morphological changes and increased neutrophil infiltration in the intestinal wall. This model exhibited upregulation of pro-inflammatory genes such as interleukin-1 beta (IL1b) and tumor necrosis factor alpha (TNFα), alongside anti-inflammatory genes like transforming growth factor beta (TGFβ) and interleukin-10 (IL10) [142]. These findings underscore the relevance of zebrafish in studying the inflammatory processes characteristic of IBD.

Recent studies have underscored the utility of zebrafish as a model organism for the investigation of inflammatory bowel disease (IBD), demonstrating their capacity to replicate essential features of the condition. Researchers have established that zebrafish exhibit gut inflammation analogous to that observed in human IBD, thereby offering a crucial platform for revealing the underlying mechanisms of the disease. A critical aspect of these investigations is the interaction between zebrafish and pathogenic bacteria, notably Adherent-Invasive *Escherichia coli* (AIEC). AIEC is recognized for its role in the pathogenesis of IBD by exacerbating inflammation and compromising the gut barrier. In zebrafish models, researchers have documented the adherent and invasive of AIEC toward intestinal cells, which elicit inflammatory responses that are reminiscent of those found in human patients [143, 144].

These findings highlight the efficacy of zebrafish in modeling IBD, enabling scientists to investigate host-pathogen interactions, immune responses, and potential therapeutic interventions within a living organism. The transparent nature of zebrafish embryos further facilitates real-time imaging of these processes, yielding visions into the dynamics of gut inflammation and the effectiveness of treatments aimed at modulating the immune response and bacterial interactions, which contribute to a deeper understanding of IBD mechanisms and the development of novel therapeutic strategies.

As research continues to advance, the application of the zebrafish model in IBD research is anticipated to broaden. Future studies may concentrate on the genetic underpinnings of IBD by employing CRISPR-Cas9 technology to generate specific zebrafish mutants that replicate human genetic variants associated with the disease. Furthermore, the investigation of new therapeutic agents, including biologics and small molecules, can be streamlined through high-throughput screening methods in zebrafish larvae. Despite their poikilothermal nature, zebrafish share considerable metabolic characteristics with humans, rendering them particularly suitable for exploring metabolic diseases and assessing potential therapeutic interventions [145].

Integrating multi-omics approaches, including transcriptomics and metabolomics, with zebrafish models augments our comprehension of the molecular pathways involved in inflammatory bowel disease (IBD). This comprehensive strategy enables researchers to investigate genetic, molecular, and imaging techniques, thereby revealing intricate networks that contribute to disease onset and progression. Multi-omics studies have identified novel biomarkers for Crohn’s disease (CD) and Ulcerative colitis (UC), facilitating differentiation between disease subtypes and severity [146]. Furthermore, research has underscored aberrant lipid metabolism in UC, identifying phospholipase A2 Group IIA (PLA2G2A) as a significant biomarker correlated with immune cell types and disease severity [147]. Multi-omics phenotyping has also characterized molecular divergences in IBD, linking serum profiles to disease activity and treatment responses [148]. Zebrafish models enable the study of IBD mechanisms, allowing for real-time imaging and genetic manipulation, which can validate findings from human studies [149]. While multi-omics approaches yield valuable insights, challenges persist in translating these findings into clinical practice, particularly concerning the complexity of IBD and individual patient variability.

In summary, the adult zebrafish model, together with various transgenic lines, provides a robust platform for investigating the mechanisms underlying colitis and other forms of IBD. The ability to manipulate genetic and environmental factors in zebrafish enhances our understanding of these complex diseases and may lead to novel therapeutic strategies. As research continues to progress, zebrafish are poised to assume an increasingly critical role in elucidating the pathophysiology of IBD and developing effective treatments. The unique advantages of this model organism, combined with innovative research techniques, hold significant promise for improving outcomes for individuals suffering from IBD.

### Liver diseases

Fatty liver disease, particularly non-alcoholic fatty liver disease (NAFLD), is recognized as the most prevalent liver disorder globally. It is estimated that approximately one billion individuals worldwide are affected by NAFLD, rendering it a significant public health concern. The initial step in the pathogenesis of NAFLD is the accumulation of lipid droplets within hepatocytes, a condition known as steatosis. This accumulation can progress through a series of stages, including hepatocellular injury, inflammation, fibrosis, cirrhosis, and ultimately liver dysfunction [150].

NAFLD encompasses a spectrum of liver conditions, ranging from simple steatosis (non-alcoholic fatty liver, NAFL) to non-alcoholic steatohepatitis (NASH), characterized by inflammation and cellular injury, which potentially lead to advanced fibrosis and cirrhosis. The progression from steatosis to more severe forms of liver disease is influenced by various factors, including obesity, insulin resistance, and metabolic syndrome [151]. Interestingly, while hepatocytes can utilize these lipid droplets as an energy source, this metabolic adaptation can paradoxically lead to further forms of hepatocyte dysfunction, thereby emphasizing the complex interplay between lipid deposition and metabolic responses [152].

### The utility of zebrafish in liver disease research

Research has demonstrated that the liver pathologies observed in zebrafish larvae closely resemble those found in mammalian systems, suggesting conserved molecular and cellular pathways involved in liver disease [82, 153]. A significant advantage of the zebrafish model is the ease with which larvae can be exposed to various hepatotoxins, such as ethanol, which induce liver disease. This accessibility enables researchers to explore the underlying mechanisms and pathogenesis of liver injury within a tractable system [7].

Other studies have investigated the effects of tunicamycin (Tm) and ethanol (EtOH) exposure on hepatocyte and biliary function in zebrafish. The findings revealed that not all hepatocyte functions were uniformly disrupted; for instance, while ethanol exposure primarily affected lipid metabolism—leading to exacerbated steatosis and impaired lipid export—tunicamycin specifically inhibited protein synthesis and secretion by disrupting N-linked glycosylation. This observation underscores the cellular stress responses that can differentially impact hepatocyte functionality [154]. Despite these disruptions, zebrafish hepatocytes retained the ability to utilize accumulated lipid droplets for energy, indicating a degree of metabolic adaptation even in the context of steatosis. Moreover, basic cellular functions, such as cell viability, remained largely intact, suggesting that while specific pathways were compromised, the overall hepatocyte metabolic machinery could still function effectively [155].

Zebrafish have emerged as a significant animal model for studying liver regeneration, particularly the role of liver progenitor cells (LPCs). These cells are pivotal for liver recovery, especially when hepatocyte proliferation is compromised due to injury. Zebrafish models facilitate the examination of LPC activation and differentiation, as they possess a remarkable capacity for recovery and permit for easy genetic manipulation. Recent studies have elucidated the complex molecular mechanisms governing LPC activation, including key signaling pathways such as YAP and mTORC1, along with influential role of inflammatory signals. The ability to visualize and manipulate these processes in zebrafish provides crucial understanding into the roles of LPCs in both regeneration and the progression of liver diseases, such as fibrosis and cancer. As research continues, zebrafish present a promising platform for identifying therapeutic targets aimed at enhancing LPC differentiation into functional hepatocytes, ultimately contributing to improved treatment strategies for patients with advanced liver disease [156].

### Investigating autosomal dominant polycystic liver disease (ADPLD)

Beyond non-alcoholic fatty liver disease (NAFLD), zebrafish models have been employed to investigate autosomal dominant polycystic liver disease (ADPLD), a condition resulting from mutations in genes such as Sect. 63 and prkcsh, which lead to the formation of liver cysts [157]. Bogert et al. (2013) developed zebrafish models harboring mutations in these genes to explore the mechanisms underlying hepatic cystogenesis. The resultant cyst formation in zebrafish closely parallels human pathology, showing this model a unique platform for testing potential therapeutic interventions. These mutations disrupt the normal growth and secretion of cholangiocytes, which are critical for liver function and cyst formation. This research highlights the significance of the zebrafish model in elucidating the genetic and cellular mechanisms implicated in polycystic liver diseases [158].

### Insights into immune interactions and disease mechanisms

Recent research conducted by Huang et al. (2024) has illuminated the interactions between hepatocytes and immune cells in the context of NAFLD, underscoring the significance of immune regulation in the progression of liver disease. The study emphasized that alterations in cellular composition and immune responses can profoundly impact influence the pathogenesis of liver disorders. Additionally, zebrafish have facilitated the exploration of various liver diseases by allowing the induction of liver damage through genetic manipulation or exposure to chemical toxins, thereby enabling researchers to investigate disease mechanisms with high fidelity [159].

The unique transparency and rapid lifecycle of zebrafish make them an ideal model for high-throughput drug screening and the identification of therapeutic candidates, proving essential for studying the cellular composition, immune environment, and genetic factors involved in liver diseases such as NAFLD and ADPLD. By leveraging the strengths of the zebrafish model, researchers can gain deeper clarity into disease progression and assess potential treatments, ultimately enhancing our understanding and management of these complex liver disorders [55, 160].

### Kidney diseases

Zebrafish (*Danio rerio*) have emerged as a robust model organism for investigation of various kidney diseases, owing to their genetic tractability, rapid developmental processes, and the capacity to observe organogenesis in real-time. Researchers have utilized diverse manipulation techniques to explore conditions such as polycystic kidney disease (PKD), acute kidney injury, glomerular disease, tubular function, and kidney regeneration [124]. The application of zebrafish models provides unique visions into the genetic and developmental mechanisms underlying these conditions, thereby enhancing our understanding of kidney pathophysiology and potential therapeutic strategies.

### Polycystic kidney disease (PKD)

Polycystic kidney disease (PKD) is a genetic disorder characterized by the abnormal dilation of kidney tubule lumens due to excessive epithelial cell proliferation. This phenomenon leads to the formation of fluid-filled cysts, which can disrupt normal kidney function, ultimately resulting in kidney fibrosis and renal failure as the cysts increase in size and number [161]. Zebrafish have proven particularly useful in investigating the genetic basis of PKD. Researchers have identified several recessive mutations that induce the development of cystic pronephroi, which are the primitive kidneys in zebrafish, thereby demonstrating their capacity to closely mimic human kidney pathology.

Key studies have shown that these mutations result in the formation of glomerular or tubular cysts, affecting critical stages of nephrogenesis such as nephron patterning [137]. For instance, specific mutations have been linked to defects in genes essential for the maintenance of tubular integrity and lumen size. In one study, twelve genes were identified in zebrafish that cause pronephric cysts, with two of these genes also associated with human cystic kidney diseases, thereby underscoring the translational potential of zebrafish research [162].

The preservation of appropriate lumen size and epithelial cell morphology is regulated by multiple signaling pathways, and disruptions in these pathways can participate cyst formation. This complexity is evidenced by studies indicating that the proper functioning of the epithelial cells lining the tubules is crucial for maintaining kidney architecture and function [163].

### Mechanisms of cyst formation

Despite significant advancements in the understanding of polycystic kidney disease (PKD), the precise mechanisms that underlie cyst formation remain only partially elucidated. Recent findings have identified multiple genes associated with the condition, particularly those involved in signaling pathways that govern cellular proliferation and differentiation. The Wnt5a protein, for instance, has emerged as a crucial regulator during the elongation of renal tubular cells via the planar cell polarity (PCP) signaling pathway. This pathway is integral to the orientation of cell division, and documented defects in PCP signaling are known to precipitate the development of kidney cysts.

In a targeted investigation, researchers performed knockdown experiments of the wnt5a gene in transgenic zebrafish (Tg(wt1b: GFP)), leading to observable phenotypic deformations, including a curly tail and the formation of kidney cysts. These findings imply that the Wnt5a-mediated PCP pathway plays an essential role in regulating tubular cell elongation and division; its disruption contributes to the characteristic cystic changes associated with PKD [164].

### Insights into glomerular diseases and podocyte biology

Zebrafish models have also yielded significant perceptions into glomerular diseases, particularly through studies focusing on podocytes, specialized cells that are critical to the kidney’s filtration system. Research has demonstrated the utility of zebrafish larvae in studying the podocyte biology, providing new perspectives on glomerular diseases and potential therapeutic targets for podocyte-associated glomerulopathies [165]. The capacity to observe dynamic processes in zebrafish larvae using advanced imaging techniques has greatly enhanced our understanding of kidney diseases, enabling researchers to visualize the effects of various genetic and environmental factors on podocyte function and integrity.

Recent studies have identified homologous genes in zebrafish that correlate with human cystic kidney diseases, further substantiating the relevance of the zebrafish model in renal research [166]. These findings are pivotal for understanding the genetic underpinnings of kidney disorders and for identifying potential therapeutic targets. For example, investigations into the signaling pathways implicated in podocyte function have facilitated the identification of novel drug candidates aimed at treating glomerular diseases.

In conclusion, the zebrafish model has proven to be an indispensable tool in the study of kidney diseases, particularly in elucidating the genetic and developmental mechanisms underlying conditions such as PKD and glomerular diseases. The visions gained from zebrafish research not only enhance our comprehension of kidney pathologies but also pave the way for innovative therapeutic approaches. By continuing to leverage the unique attributes of zebrafish, researchers can further unravel the complexities of kidney disorders, ultimately advancing our understanding and management of these debilitating conditions.

## Conclusions

Zebrafish are increasingly recognized as ideal models for studying various human diseases due to their genetic similarities and functional conservation with humans, particularly in the kidney, liver, and Gastrointestinal systems. Their ability to develop transgenic strains rapidly and undergo high-throughput chemical screening allows researchers to observe epithelial cell behavior across different organs. This small fish species has been instrumental in exploring genetic diseases and cancer, with advanced imaging techniques providing understandings of cellular mechanisms that can inform the development of effective therapeutics.

Despite their advantages, zebrafish have limitations in modeling diseases related to organs or tissues that they lack, such as the prostate or lungs. However, their shared functionalities with mammals in organs like the liver and kidney make them suitable for investigating related diseases. Innovations like the Casper transgenic line address challenges such as transparency in adult zebrafish, enabling non-invasive visualization of organs and cells. Overall, zebrafish offer a practical and influential platform for studying epithelial tissue diseases, and continued investment in this research is essential for enhancing our understanding of disease mechanisms and pathogenesis.

## Data Availability

Not applicable.

## Abbreviations

ADPLD: Autosomal Dominant Polycystic Liver Disease
AIEC: Adherent-Invasive Escherichia coli
ALD: Alcoholic Liver Disease
ALR: Augmenter of Liver Regeneration
ALT: Alanine Aminotransferase
AST: Aspartate Aminotransferase
DALDA: D-Arg2,Lys4]dermorphin-(1,4)-amide
dpf: Days post-fertilization
EGFP: Enhanced Green Fluorescent Protein
ER: Endoplasmic reticulum
hpf: Hours post-fertilization
I FABP: Intestinal fatty acid-binding protein
IL: 1b Interleukin-1b
IL-10: Interleukin-10
GFP: Green Fluorescent Protein
HCC: Hepatocellular Carcinoma
IBD: Inflammatory Bowel Disease
MO: Morpholino antisense oligonucleotide (MO)
MOR: µ-opioid receptor
MPO: Myeloperoxidase
NAFLD: Non-Alcoholic Fatty Liver Disease
NSAID: Non-Steroidal Anti-Inflammatory Drug
PCP: Planar Cell Polarity
PKD: Polycystic Kidney Disease
PLD: Polycystic Liver Disease
TNBS: Hapten 2,4,6-trinitrobenzene sulfonic acid
TNF: Tumor Necrosis Factor
UPR: Unfolded Protein Response
Tm: Tunicamycin
